# Neurodevelopmental assessment of HIV-exposed uninfected and early-treated HIV-infected children: study protocol

**DOI:** 10.1186/s13104-018-3331-8

**Published:** 2018-04-06

**Authors:** Renate Strehlau, Tamryn van Aswegen, Joanne Potterton

**Affiliations:** 1 Empilweni Services and Research Unit, Rahima Moosa Mother and Child Hospital, Department of Paediatrics and Child Health, Faculty of Health Sciences, University of the Witwatersrand, Johannesburg, South Africa; 2 Department of Physiotherapy, School of Therapeutic Sciences, Faculty of Health Sciences, University of the Witwatersrand, Johannesburg, South Africa

**Keywords:** Early childhood development, HIV, Early antiretroviral treatment

## Abstract

**Objective:**

Sub-Saharan Africa has the highest prevalence of children at risk of not achieving their developmental potential, attributable largely to the human immunodeficiency virus (HIV) pandemic coupled with negative environmental factors. Childhood developmental stimulation programmes can mitigate adverse outcomes.

**Methods:**

Neonates testing HIV positive at birth will be initiated on antiretroviral treatment (ART) and receive an age-appropriate stimulation program, updated at 3 monthly intervals through the first year of life. Neurodevelopment at 12 months of age will be assessed using the Bayley Scales of Infant and Toddler Development, Third Edition (BSID-III). Outcomes will be compared with HIV-infected and HIV-exposed uninfected children (HEU) not having received the stimulatory intervention. Associations between neurodevelopmental outcomes, environmental factors, and parental stress will be investigated. The study will take place at a single site in Johannesburg, South Africa. This non-randomised controlled intervention study, with a single non-blinded comparative intervention group, aims to investigate whether an early childhood stimulation programme used in conjunction with ART initiated at birth can positively impact neurodevelopmental outcomes at 1 year of age in children infected with HIV.

*Trial registration* 15 January 2018, Pan African Clinical Trial Registry PACTR201801002967587

## Introduction

Worldwide an extraordinary number of children are at risk of not developing their full potential. Using poverty and stunting as proxy markers for estimating children at risk of early childhood developmental (ECD) delays, a 2010 Lancet Global Health report estimated that 249.4 million children (43% of all children in 2010) under the age of 5 years living in low- and middle-income countries (LMIC) were at risk of not reaching their developmental potential [1]. Sub-Saharan Africa was reported as having the highest prevalence of children at risk—66% in 2010. The HIV pandemic in South Africa contributes to this statistic with 240,000 (210,000–260,000) children under 15 years of age infected with HIV [2].

The human immunodeficiency virus is neurotropic and invasion of the developing infant brain results in significant negative neurodevelopmental consequences [3]. Reported developmental shortfalls include delays in motor, cognitive, and language development [4]. Careful assessment of the child’s developmental abilities is important as children may continue to acquire new skills, but function consistently below the age-related developmental norm on standardised assessments [5]. The severity of the neurological deficit is increased relative to the degree of immunosuppression, stage of HIV disease [6], and timing of ART initiation. ART started at a young age is associated with improved neurocognitive outcomes [7, 8]. However, despite suppressive ART, neurodevelopmental delays—which may at times be quite subtle—continue to persist into childhood [9] raising concern for the adolescent years [10].

Data describing developmental trajectories of HEU children is conflicting with some studies reporting cognitive and motor deficits [11], but these may also be attributed to co-varying risks related to parental HIV infection such as poverty, disruption in schooling, and reduced wellbeing [12].

Effective interventions stimulating ECD can have far-reaching positive outcomes, but little is known about the long-term impact of an intervention started at birth in conjunction with early antiretroviral treatment (ART) initiation. The primary aim of this study is to assess neurodevelopmental outcomes of early-treated HIV-infected children receiving an age-appropriate stimulation program and compare them with HIV-infected and HEU children not having received the stimulatory intervention. In addition, associations between neurodevelopmental outcomes, environmental factors, and parental stress will be examined.

## Main text

The study is designed as a non-randomised controlled intervention study.

### Subject selection

Study participants will be recruited from an existing cohort of early-treated HIV-infected and HEU children enrolled onto a clinical trial investigating viral latency and early neonatal provision of anti-retroviral drugs (NCT02431975). Children enrolled and in follow-up in the existing cohort will be offered inclusion into this study, making up the assessment only group. New-born infants will be offered co-enrolment onto the existing study and will make up the group receiving the intervention as well as the assessment. Refusal to participate in one protocol will not jeopardise inclusion onto the other. The trial will be conducted at Rahima Moosa Mother and Child Hospital (RMMCH), Johannesburg, South Africa. The study was approved by the Human Research Ethics Committee of the University of the Witwatersrand (M170653) and registered with the Pan African Clinical Trial Registry (PACTR 201801002967587). Caregiver written informed consent will be obtained.

### Sample size

All children in the existing cohort will be enrolled and assessed using the BSID-III at a single time-point i.e. either at 12, 24 or 36 months of age. 45 children will be enrolled into the intervention group. Hutchings and Potterton, investigating developmental outcomes of HIV-infected and HEU infants [13] reported average cognitive composite scores of 100.31 ± 12.75 for HEU and 75.89 ± 17.69 for HIV-infected infants. Groups of 45 will provide > 90% power to detect a mean cognitive composite score difference of 25 assuming a standard deviation of 17. Hutchings also reported a mean composite language score of 96.28 ± 10.13 and 77.18 ± 16.22 and mean composite motor score of 106.16 ± 10.20 and 79.71 ± 20.91 for HEU and HIV-infected groups respectively. Groups of 45 participants provide > 90% power to detect a difference of 25 in the mean language and motor scores assuming a standard deviation of 16 and 20, respectively. Feasible study enrolment period; number of births per month at RMMCH; and maternal and neonatal HIV prevalence were considered when planning the sample size.

### Inclusion and exclusion criteria

Children less than 36 months enrolled onto the existing cohort trial are eligible for inclusion. Newborn HIV-infected infants initiated on ART whose caregiver provides informed consent will be included. Caregivers unable to comply with follow-up; serious birth defects; uncontrolled maternal psychiatric illness; and inability to provide informed consent will result in study exclusion.

### Study procedures

Trained assessors will conduct the BSID-III on children enrolled from the existing cohort at a single time point. Inter-rater reliability of BSID-III administration will be established. Prematurity will be corrected until 24 months chronological age for a gestational age < 37 completed weeks.

The BSID is a direct assessment test using test kit objects to evoke a response. Five developmental domains are evaluated: cognitive, motor, language, social-emotional and adaptive behavior [14]. The BSID have been normed in South Africa [15].

The intervention group will receive a standardised set of developmentally stimulating toys. At three-monthly intervals, additional items will be provided (Table 1).

**Table 1:** Items provided to the intervention group through the first year of life

Child’s age	Items provided
Birth	Baby blanket, washcloth, booties and hat, rattle
3 months	Mirror, small ball, book, squeaky toy
6 months	Ring stacker, large ball
9 months	Wooden blocks, stacking cups, book
12 months	Wooden form board, toy car, plastic doll, picture book, crayons and paper

Caregiver information cards developed in South African as part of a developmental activity programme will be provided three-monthly [16]. The card explains what is expected of a child at certain ages in terms of physical, cognitive, language and socio-emotional development. Play ideas and indications of possible developmental delay are included.

BSID-III will be conducted on the intervention group at 12 months of age. The intervention includes visible components—educational toys and parental information cards—and clinic files of these children will be marked to ensure they receive the intervention. Consequently, neither study participants nor assessors are masked to the intervention assignment. Furthermore, by nature of the timing of the assessment conducted at the 12 month follow-up visit the assessor would be able to deduce that the child received the intervention. Assessors have been trained to complete the assessment regardless of exposure to developmental interventions. Baseline comparability in the HIV-infected infants will be established.

### Study measures

Demographic and health-related variables will be collected from the patient’s files for participants enrolled in the existing cohort. Infant data includes birth history; serial anthropometric data; morbidity data; ART history; concurrent medication; immunisations; feeding history; and laboratory data i.e. HIV viral load, HIV total nucleic acid test, CD4 count, and full blood count (FBC). Maternal data consists of: serial anthropometric measurements; ART history; and laboratory data i.e. HIV viral load, CD4 count, and FBC. Permission for data use has been granted by the database holder and site clinical staff are aware of the aims and logistics of the study.

Composite scores for the three developmental subscales of the BSID-III which are administered with direct child interaction—cognitive, motor, and language—will be recorded [14]. Raw scores are converted to scaled scores which are used to derive the composite score for each subscale.

The household survey and the Parenting Stress Index-Short Form (PSI-SF) will be completed with the child’s caregiver. The household survey is a self-styled questionnaire gathering data about the household in which the child lives. Questions cover the following categories: family structure; number of household members diagnosed with HIV; parental nationality; employment; cigarette and alcohol use; water and sanitation measures; type of housing; exposure to sexual abuse and physical violence; childcare arrangements; and access to electricity and basic electronic household items. The household survey consists of a broad set of variables as ECD is influenced both negatively and positively by a myriad of factors [17–19]. The PSI-SF is the condensed version of a self-reported screening tool used to explore parental stress levels associated with parenting children aged 1 month–12 years [20]. Maternal HIV infection can impact parenting skills which in turn influences the child’s wellbeing, ECD and childhood outcomes [21, 22].

### Data analysis

De-identified data from paper case report forms will be entered into a secure database. Statistical calculations will be performed using SAS version 9.4 (Cary, North Carolina, USA). Anthropometric Z-scores will be calculated using WHO software [23]. P-values will be two-tailed and p-values < 0.05 will be considered statistically significant.

Descriptive statistics will describe the characteristics of the early-treated perinatally HIV-infected children enrolled in the existing cohort in terms of demographics, growth parameters, and HIV-related factors. Composite cognitive, language and motor scores will be described. Continuous variables with normal distribution will be described using means and standard deviation with a 95% confidence interval. Frequency tabulations and percentages will be used to describe categorical variables. “At risk” of developmental delay will be defined as any composite score less than 85, and “delayed” as a score less than 70 [24]. Number of children in these two categories will be explored and associations between patient and disease characteristics investigated using the Chi squared test at 5% level of significance. Mean composite scores will be compared between children assessed at 12, 24 or 36 months of age using ANOVA.

Mean composite scores and number of children “at risk” or “delayed” in each subscale will be compared between early-treated HIV-infected and HEU infants assessed at 12 months of age. T tests will compare demographic, clinical and anthropometrical data between groups. The analysis will be expanded to include the HIV-infected infants having received the ECD intervention. The Chi squared test and Kruskall-Wallis 1-way ANOVA will be used to measure variable difference between groups.

Univariate and multiple regression analyses will be performed to establish associations with poor outcomes on the three subscales i.e. composite scores less than 70 or 85. Variables used to establish associations with poor outcomes will include maternal antenatal and postnatal health-related factors, infant birth data, infant health and HIV-related variables, and data pertaining to socioeconomic status and parental stress. Logistic regression will be conducted on various risk factors for each subscale and a score of ≤ 85 will constitute a poor outcome.

### Conclusion

HIV puts early neurodevelopment of HIV-infected and HEU children at risk [4, 11]. ECD interventions should form part of the management of HIV-infected and HEU children [25]. We will compare neurodevelopmental outcomes of early-treated HIV-infected children receiving an age-appropriate stimulation program with early-treated HIV-infected and HEU children not receiving an intervention.

## Limitations

Although comprehensive, the household survey may not include all confounding variables as development is influenced by innumerable factors. Children in the HEU control group would be exposed to the same confounders as the HIV-infected infants. Use of toys and materials is caregiver dependent—children may not be given the intervention in the home environment. Use of the intervention depends on parental acceptance in accordance with cultural norms and child rearing practices. Use of the intervention will be carefully explained to the caregiver and the caregiver will be encouraged to initiate play and interact with her infant. Sufficient time will be allowed for answering questions and repeating explanations. Study attrition will reduce the planned cohort size. Caregivers returning at incorrect study time points will result in children missing the ideal BSID assessment age. The following measures will be undertaken to maintain the cohort in follow-up. Caregivers will be provided with appointment cards for follow-up visits, and study visits will be scheduled for a mutually convenient day. Caregivers will receive a telephone call to remind them of their study visit the day before the visit is due. Visits will be rescheduled if caregivers are unable to honour their appointments. Completion of the assessment depends on the availability of trained assessors. Two assessors have been trained to ensure an assessor is available when scheduling appointments. Assessment bias may occur as assessors have not been blinded to the intervention group; however, assessors have been trained to conduct the assessment in a standardised manner for all participants.

## Abbreviations

ART: antiretroviral treatment
BSID-III: Bayley Scales of Infant and Toddler Development, Third Edition
ECD: early childhood development
FBC: full blood count
HEU: HIV exposed uninfected
HIV: human immunodeficiency virus
LMIC: low- and middle-income countries
PSI-SF: Parenting Stress Index-Short Form
RMMCH: Rahima Moosa Mother and Child Hospital
